# Percutaneous Versus Surgical Femoral Cannulation in Endoscopic Minimally Invasive Heart Valve Surgery: A Single-Centre Retrospective Comparison of Cut-Down, ProGlide and MANTA Closure Strategies

**DOI:** 10.3390/jcm15155999

**Published:** 2026-08-01

**Authors:** Ahmed Ghazy, Mohamad Albitar, Edoardo Zancanaro, Daniel-Sebastian Dohle, Katja Buschmann, Hendrik Treede

**Affiliations:** Department of Cardiac and Vascular Surgery, University Medical Centre, Johannes Gutenberg-University Mainz, 55131 Mainz, Germany

**Keywords:** minimally invasive cardiac surgery, femoral cannulation, vascular closure device, ProGlide, MANTA, endoscopic surgery

## Abstract

**Background/Objectives**: Femoral cannulation is the cornerstone of endoscopic minimally invasive heart valve surgery (MICS). We compared three femoral access strategies—surgical cut-down, suture-based percutaneous closure (ProGlide^®^) and plug-based percutaneous closure (MANTA^®^)—for access-site outcomes, operative times and 30-day morbidity. **Methods**: This is a retrospective single-centre analysis of 445 consecutive patients undergoing endoscopic MICS (February 2021–May 2025). Access strategy (cut-down *n* = 119; ProGlide *n* = 219; MANTA *n* = 107) was selected by preoperative CT angiography and the surgeon’s discretion, with hostile anatomy triaged to cut-down. Baseline characteristics, intraoperative timing, primary haemostasis, conversion and 30-day outcomes were compared; a multivariable model within the percutaneous cohort adjusted for arterial cannula size and baseline covariates was used. **Results**: The mean age was 61.6 ± 11.3 years; 60.8% were male. EuroSCORE II was comparable between the percutaneous and cut-down groups (1.89 ± 2.55 vs. 2.03 ± 2.41; *p* = 0.594). Primary haemostasis was higher with MANTA than ProGlide (96.2% vs. 83.1%; *p* = 0.001; adjusted OR 4.79). Conversion to cut-down was similar (3.7% vs. 3.6%; *p* = 1.000). Intervention-requiring groin complications were fewer with percutaneous access (3.7% vs. 7.5%; *p* = 0.232). Cross-clamp, bypass and total operative times were shortest with MANTA (all *p* < 0.001). In-hospital mortality was 2.1% vs. 1.6% (*p* = 1.000). **Conclusions**: In appropriately selected patients, percutaneous femoral cannulation in MICS is safe and is associated with fewer groin complications and shorter operative times than cut-down. Within the percutaneous arm, MANTA was associated with a higher rate of primary haemostasis in our institutional experience. Cut-down remains indispensable for hostile femoral anatomy.

## 1. Introduction

Endoscopic minimally invasive heart valve surgery (MICS) has evolved from an investigational technique to a standard-of-care procedure for selected patients with mitral, tricuspid and aortic valve disease [1,2]. Compared with conventional sternotomy, endoscopic approaches reduce surgical trauma, accelerate recovery and improve patient satisfaction without compromising long-term outcomes [3,4,5]. The German Heart Surgery Report documents that more than 75% of isolated valve operations in Germany are now performed using minimally invasive access [6].

A safe and reproducible femoral cannulation strategy is the cornerstone of every endoscopic MICS procedure, since cardiopulmonary bypass is established peripherally rather than centrally. Three principal strategies are in current clinical use: surgical cut-down with primary vascular repair, suture-based percutaneous closure with the Perclose ProGlide^®^ system (Abbott Vascular, Santa Clara, CA, USA) and plug-based percutaneous closure with the MANTA^®^ device (Teleflex, Wayne, PA, USA) [7,8,9]. Each strategy carries a distinct profile of advantages and limitations. Cut-down provides direct vascular control and is reliable in calcified or small-calibre vessels but adds local soft-tissue trauma, prolongs operative time and is associated with lymphatic fistulae and wound complications [10,11]. Percutaneous strategies minimise soft-tissue dissection and are well established in the trans-femoral TAVI literature [12,13]. However, randomised data comparing plug-based with suture-based closure derive almost exclusively from TAVI cohorts, and the findings cannot be extrapolated uncritically to the cardiac surgery setting, given the differences in age, sex and atherosclerotic burden [14,15].

Direct comparative evidence in surgical patients undergoing MICS remains limited [16,17,18]. We therefore performed a single-centre retrospective analysis of 445 consecutive patients who underwent endoscopic MICS at our institution between February 2021 and May 2025. Our objective was to compare the three principal femoral access strategies (cut-down, ProGlide and MANTA) with respect to (i) primary haemostasis at the puncture site, (ii) conversion to surgical cut-down, (iii) intervention-requiring groin complications, (iv) intraoperative timing and (v) 30-day mortality and major morbidity.

## 2. Materials and Methods

### 2.1. Study Design and Patient Population

We conducted a retrospective single-centre cohort study at the Department of Cardiac and Vascular Surgery, University Medical Centre Mainz, Germany. All consecutive adult patients (≥18 years) who underwent 3D endoscopic MICS via right antero-lateral mini-thoracotomy between February 2021 and May 2025 were screened for inclusion. The inclusion criteria were (i) elective or urgent endoscopic minimally invasive valve surgery (mitral, tricuspid, aortic or combined) and (ii) femoral arterial and venous cannulation for cardiopulmonary bypass. The exclusion criteria were (i) a full sternotomy approach, (ii) emergency surgery for haemodynamic instability and (iii) re-do MICS in which a non-femoral cannulation strategy (e.g., axillary) was used.

### 2.2. Femoral Access Strategies

Patients were stratified by femoral access strategy into three groups: (1) surgical cut-down (*n* = 119), (2) percutaneous suture-based closure with two ProGlide devices used in the pre-closure technique (*n* = 219) and (3) percutaneous plug-based closure with a single MANTA device (*n* = 107). Strategy selection was at the discretion of the attending surgeon based on preoperative computed tomography (CT) angiography of the iliofemoral axis and bedside ultrasound. Cut-down was preferred when the imaging showed marked calcification, small calibre (<6 mm), severe tortuosity or significant peripheral arterial disease. The Seldinger technique was used for all percutaneous punctures, performed under ultrasound guidance of the correct puncture height above the femoral bifurcation [19,20].

### 2.3. Operative Technique

All procedures were performed by experienced MICS surgeons via a 4–6 cm right antero-lateral mini-thoracotomy in the fourth intercostal space. Cardiopulmonary bypass was established with retrograde femoral arterial perfusion and femoral venous return via a 17–21 Fr arterial cannula and a long venous cannula advanced into the superior vena cava under transoesophageal echocardiographic guidance. Aortic occlusion was achieved with a transthoracic Chitwood clamp. Antegrade custodial cardioplegia was administered. After valve repair or replacement, decannulation was performed using one of the three closure strategies. In the cut-down group, primary repair of the femoral artery was performed with 5–0 Prolene sutures.

### 2.4. Outcome Measures

The primary endpoint was the incidence of intervention-requiring groin complications, defined as any access-related event mandating surgical or endovascular treatment.

After device deployment, manual compression was permitted; persistent oozing or any bleeding necessitating an adjunctive device or cut-down after full heparin antagonisation with protamine was classified as failure of primary haemostasis.

Secondary endpoints included primary haemostasis (defined as effective haemostasis without any need for an adjunctive closure device or cut-down conversion); use of an adjunctive closure device; conversion to surgical cut-down; specific groin complications (lymphatic fistula/seroma, infection, intervention-requiring haematoma, pseudoaneurysm, vessel occlusion); cross-clamp, cardiopulmonary bypass and total operative times; in-hospital and 30-day mortality; re-thoracotomy for bleeding; new pacemaker implantation; and perioperative stroke.

### 2.5. Statistical Analysis

Continuous variables are presented as the mean ± standard deviation and compared via one-way ANOVA or the Kruskal–Wallis test, depending on the distribution. Categorical variables are presented as counts and percentages and compared via Chi-square or Fisher’s exact test as appropriate. Pairwise post hoc comparisons were performed via the Mann–Whitney U test for continuous data and Fisher’s exact test for categorical data. A two-sided *p*-value < 0.05 was considered statistically significant. To account for the non-random allocation of access strategy, a multivariable binary logistic regression model was constructed within the percutaneous cohort to identify independent predictors of primary haemostasis, with arterial cannula size included as a covariate together with the baseline variables that differed among groups; the results are reported as adjusted odds ratios with 95% confidence intervals. All analyses were performed using Prism 11 (GraphPad Software, Boston, MA, USA).

## 3. Results

### 3.1. Baseline Characteristics

Between February 2021 and May 2025, 445 consecutive patients underwent endoscopic MICS with femoral cannulation at our institution. The mean age was 61.6 ± 11.3 years; 271 (60.8%) were male. The percutaneous and cut-down cohorts had comparable EuroSCORE II^19^ values (1.89 ± 2.55% vs. 2.03 ± 2.41%; *p* = 0.594) and NYHA class distributions (*p* = 0.079). The cut-down group had a higher proportion of male patients (71.4% vs. 57.0%; *p* = 0.006), higher BMI (26.6 ± 5.4 vs. 25.4 ± 4.5; *p* = 0.032), atrial fibrillation (45.3% vs. 35.2%; *p* = 0.061) and extracardiac arteriopathy (15.9% vs. 9.5%; *p* = 0.063), reflecting the deliberate triage of patients with hostile femoral anatomy to surgical cut-down. Detailed baseline characteristics are summarised in Table 1.

The multivariable analysis with the percutaneous group (Manta vs. Proglide) showed, after mutual adjustment, male sex (OR 2.19, *p* = 0.021) and, weakly, EuroSCORE II (OR 0.82, *p* = 0.046). No comorbidity or anatomical risk factor (PAD, COPD, CAD, diabetes, LVEF) independently distinguished the groups. The MANTA vs. ProGlide comparison is therefore not driven by a systematically sicker or anatomically hostile cohort.

### 3.2. Operative Procedures and Intraoperative Timing

The procedural distribution is shown in Table 2. Isolated mitral valve operations were performed in 378 patients (84.9%), isolated tricuspid operations in 9 (2.0%), isolated aortic operations in 8 (1.7%) and combined valve procedures in 50 (11.2%). Concomitant left atrial appendage occlusion was performed in 66 patients (14.8%) and atrial ablation in 110 (24.7%) (Table 2b). Cross-clamp, cardiopulmonary bypass and total operative times were significantly shorter in the MANTA group, intermediate in the ProGlide group and longest in the cut-down group (all *p* < 0.001 across the three groups; Table 2a). Pairwise post hoc analyses confirmed significant differences among all three groups for cross-clamp time and total operative time.

### 3.3. Primary Access Success and Conversion

**Figure 1:**
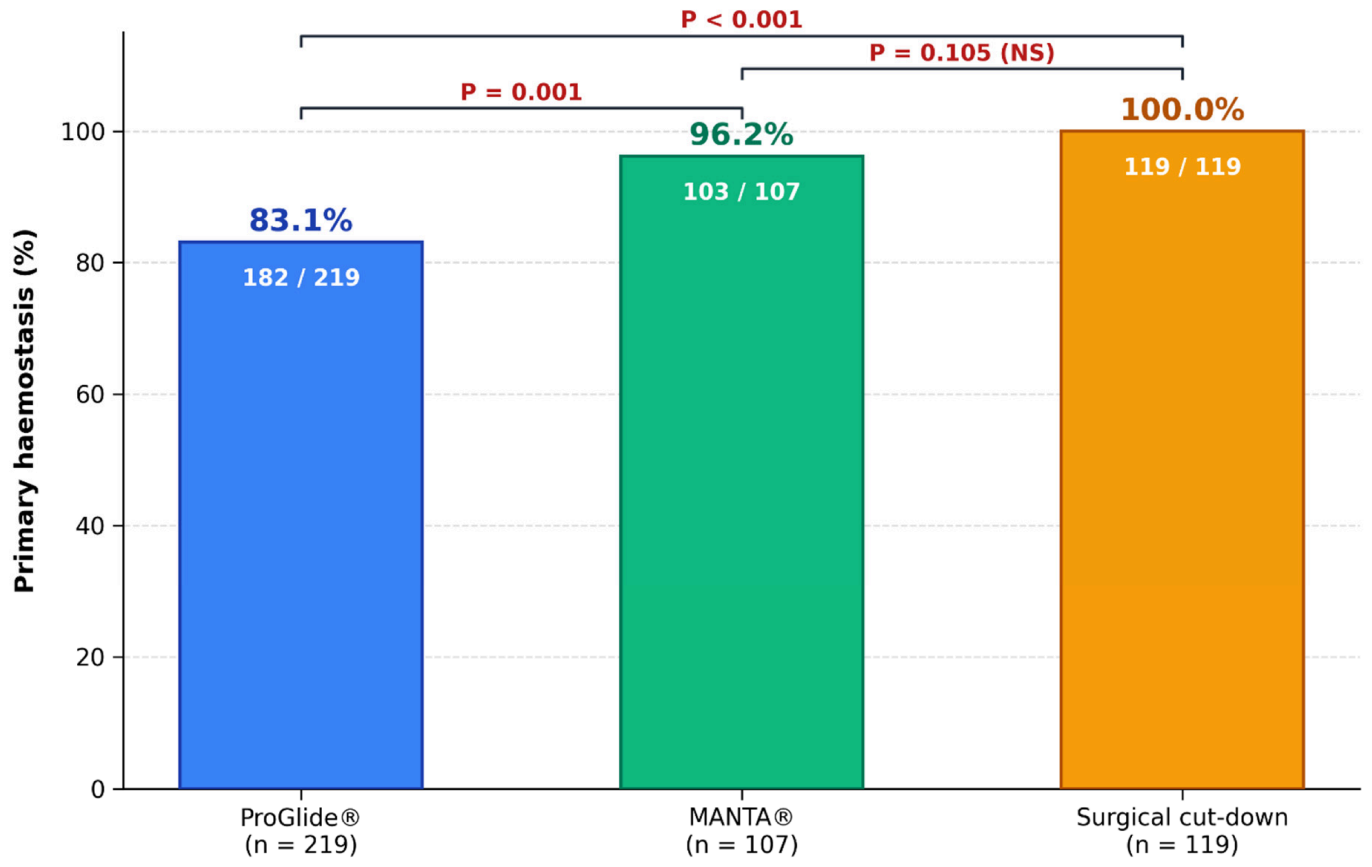
Primary haemostasis rates by closure strategy. MANTA achieved 96.2% primary haemostasis; ProGlide, 83.1%; and cut-down, 100% (*p* < 0.001 across all three groups; *p* = 0.001 for MANTA vs. ProGlide).

Primary haemostasis (effective access-site closure without the need for an adjunctive device or cut-down conversion) was achieved in 96.2% of MANTA patients (103/107), 83.1% of ProGlide patients (182/219) and 100% of cut-down patients (119/119) (*p* < 0.001 for the overall comparison; Table 3a). In a direct pairwise comparison, MANTA was associated with a higher rate of primary haemostasis than ProGlide (96.2% vs. 83.1%; *p* = 0.001), whereas the difference between MANTA and cut-down was not significant (96.2% vs. 100%; *p* = 0.105). After multivariable adjustment for cannula size in the percutaneous group (Manta vs. Proglide) (Table 3b) and baseline covariates, device type remained an independent predictor: adjusted OR = 4.79 (95% CI: 1.64–13.95; *p* = **0.004**) (Figure 1).

In the ProGlide group, 29 patients (13.2%) required adjunctive Angio-Seal^®^ placement to achieve haemostasis after a failed pre-closure strategy. Conversion to surgical cut-down for uncontrollable bleeding was required in eight ProGlide patients (3.6%) and four MANTA patients (3.7%; *p* = 1.0 between percutaneous arms).

### 3.4. Groin Complications

Intervention-requiring groin complications occurred in 12/326 patients (3.7%) in the percutaneous group and 9/119 patients (7.5%) in the cut-down group (*p* = 0.232; Table 3a). Lymphatic fistulae or seromata occurred in five patients (4.2%) in the cut-down group and in none of the percutaneous patients (*p* = 0.002). Wound infection (*n* = 1; 0.8%) and superficial haematoma requiring intervention (*n* = 1; 0.8%) were observed exclusively in the cut-down group. Conversion to surgical cut-down was the most frequent specific complication in the percutaneous arms (3.6% ProGlide vs. 3.7% MANTA).

### 3.5. Thirty-Day Mortality and Major Morbidity

**Figure 2:**
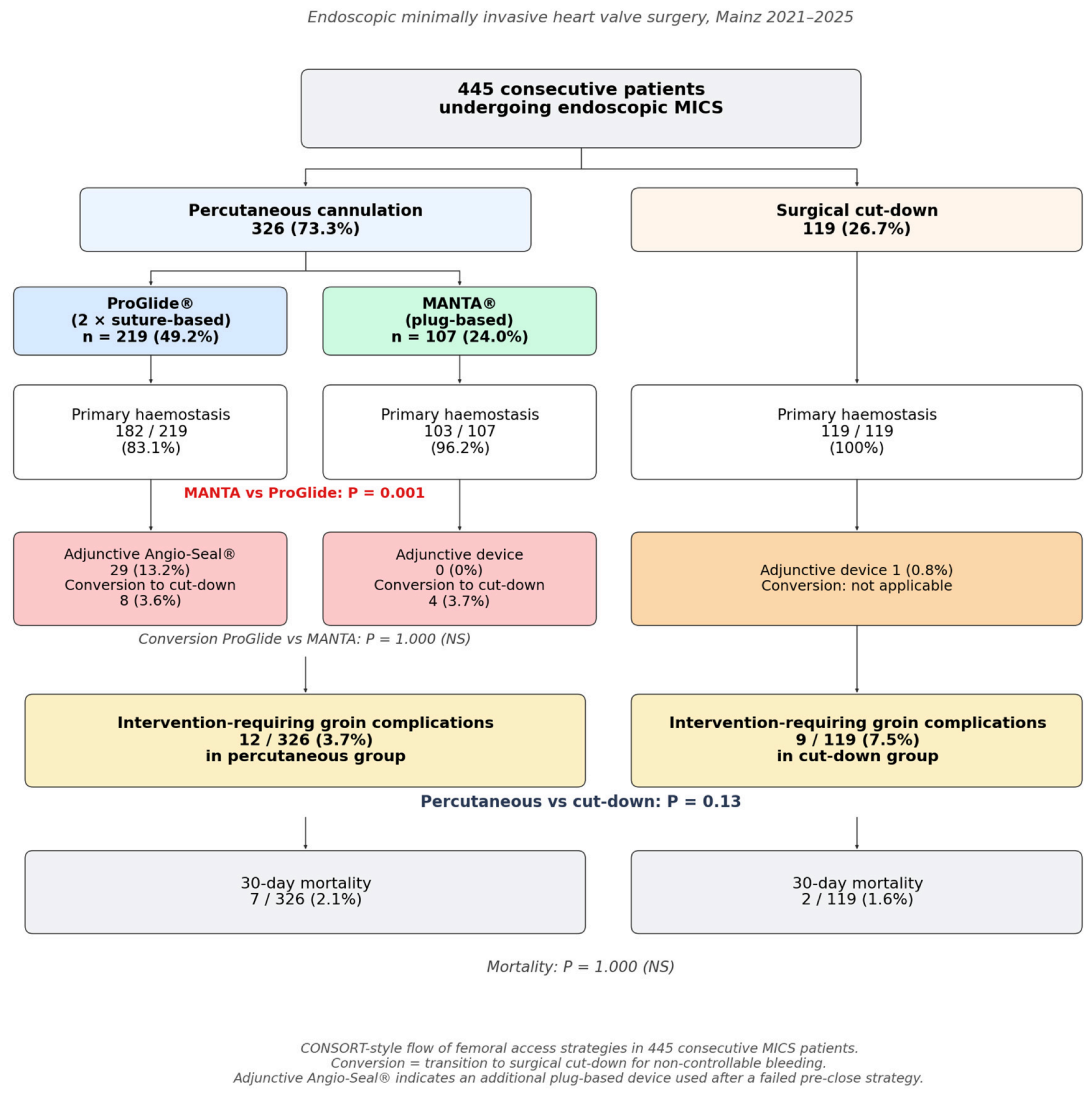
Flow diagram of the study. Of 445 consecutive patients undergoing endoscopic minimally invasive heart valve surgery between 2021 and 2025, 326 (73.3%) were managed with percutaneous femoral cannulation (ProGlide *n* = 219; MANTA *n* = 107) and 119 (26.7%) with surgical cut-down. Conversion to cut-down was required in eight ProGlide and four MANTA patients.

In-hospital and 30-day mortality were 7/326 (2.1%) in the percutaneous group and 2/119 (1.6%) in the cut-down group (*p* = 1.000). Re-thoracotomy for haematoma evacuation was required in 28 percutaneous (8.5%) and 5 cut-down patients (4.2%; *p* = 0.152). New permanent pacemaker implantation was performed in 17 percutaneous (5.2%) and 3 cut-down patients (2.5%; *p* = 0.305). Perioperative stroke and myocardial infarction were rare and did not differ significantly across groups (Table 4) (Figure 2).

## 4. Discussion

In this single-centre retrospective analysis of 445 consecutive patients undergoing endoscopic MICS, we observed three principal findings: (1) percutaneous femoral cannulation was associated with fewer intervention-requiring groin complications than surgical cut-down (3.7% vs. 7.5%; *p* = 0.232); (2) within the percutaneous arms, MANTA was associated with a higher rate of primary haemostasis than ProGlide (96.2% vs. 83.1%; *p* = 0.001), while conversion rates to cut-down were comparable (3.6% vs. 3.7%); and (3) operative times were shortest with MANTA, intermediate with ProGlide and longest with cut-down (all *p* < 0.001). In-hospital mortality was low and did not differ among strategies.

Our findings are consistent with the current literature on percutaneous cannulation in MICS. Moschovas and colleagues reported significantly lower groin complication rates with percutaneous cannulation in a single-centre study [11], while El-Sayed Ahmad and colleagues documented a marked reduction in local complications in 490 patients undergoing MANTA closure (2.2% vs. 8.1%; *p* = 0.003) [17]. Ismail and colleagues recently reported the safety of a single Perclose ProGlide technique versus cut-down, [21,22] and a multicentre registry of more than 1000 percutaneously cannulated MICS patients confirmed this trend [23]. A systematic review and meta-analysis by Kirov and colleagues concluded that percutaneous cannulation provides a more favourable safety profile in MICS overall [24].

The observed difference in primary haemostasis between MANTA and ProGlide deserves careful interpretation. Before attributing this to the device design, an important confounder must be acknowledged: the mean arterial cannula was significantly smaller in the MANTA cohort (17.7 ± 1.3 Fr) than in the ProGlide cohort (18.3 ± 1.2 Fr). Because a smaller arteriotomy is intrinsically easier to seal, part of this difference may reflect access-site calibre rather than the plug mechanism alone; we therefore included cannula size in a multivariable model (see Section 2) and report whether MANTA remained an independent predictor of primary haemostasis after adjustment. The randomised CHOICE-CLOSURE trial in 516 TAVI patients reported faster haemostasis with MANTA but a higher rate of access-site vascular complications compared with two ProGlide devices (19.4% vs. 12.0%; *p* = 0.029) [14]. The direction of this finding aligns with ours regarding primary haemostasis; however, unlike CHOICE-CLOSURE, our cohort showed no significant difference in access-site complication rates between devices. This might be due to several differences between the two settings that may account for the discrepancy. First, arterial sheath calibres are substantially smaller in TAVI (14–16 Fr) than in endoscopic MICS (17–21 Fr), so the relative challenge facing each closure system differs. Second, CHOICE-CLOSURE compared MANTA with a two-ProGlide pre-closure strategy, whereas the suture-based arm in our cohort also used two ProGlide devices but in a surgical population with different haemostatic conditions, including residual systemic heparinisation at decannulation. Third, the endpoint definitions are not interchangeable: CHOICE-CLOSURE used the VARC-2 composite of access-site vascular complications, while our primary endpoint was intervention-requiring groin complications, which is a narrower clinical construct. Together these factors caution against direct extrapolation of TAVI data to the MICS setting. A subsequent CHOICE-CLOSURE substudy identified severe femoral arterial calcification as an independent predictor of plug failure [15]. Conversely, the SAFE-MANTA pivotal study reported a low complication rate of 4.2% in 263 patients [25]. The MASH randomised pilot trial (van Wiechen et al.) and the multicentre PLUG-VS.-SUTURE registry both reported no significant differences between plug- and suture-based strategies [26,27]. The TAVI population is older, more often female in the abovementioned studies and carries a heavier atherosclerotic burden than the typical MICS cohort, which may explain why the findings cannot be extrapolated linearly. In dedicated MICS series, MANTA appears to perform favourably [17,18,28].

The mechanistic difference between the two percutaneous systems is relevant to surgical practice. Suture-based devices depend on accurate needle deployment, knot tension and vessel-wall integrity, which can be compromised under residual systemic heparinisation; the requirement for an adjunctive Angio-Seal in 13.2% of ProGlide patients in our series reflects this. Plug-based systems achieve immediate mechanical sealing through the sandwich principle of an intraluminal toggle and an extravascular collagen plug, which is less dependent on intact plasmatic coagulation [8,9]. This may explain the higher first-pass success rate with MANTA in our cohort. Operative times were also shortest with MANTA, consistent with the reduced procedural complexity at the closure step. The cut-down group had the longest cross-clamp, bypass and operative times, partly because of the dissection itself and partly because more complex combined procedures were performed in this group. Consequently, the longer cross-clamp and bypass times in the cut-down group should not be interpreted as a consequence of open femoral access, which contributes only a minor increment to the total operative time and none to ischaemic time.

Importantly, soft-tissue trauma was confined almost exclusively to the cut-down group. Lymphatic fistulae or seromata occurred in five cut-down patients (4.2%) and in none of the percutaneous patients; wound infection and intervention-requiring haematoma were also observed only after cut-down. These complications are clinically significant because they prolong hospitalisation, delay mobilisation and undermine the benefits of an otherwise minimally invasive operation. They are particularly relevant in patients with obesity, diabetes or fragile skin. The lower local burden of percutaneous strategies aligns with the principles of enhanced recovery after cardiac surgery [29,30].

Several methodological aspects underpin our results. All percutaneous punctures were performed under ultrasound guidance, in line with the FAUST and UNIVERSAL randomised trials [20,21]. Preoperative CT angiography of the iliofemoral axis was performed routinely. Patients with a hostile anatomy (severe calcification, small calibre, severe tortuosity or marked peripheral arterial disease) were preferentially assigned to surgical cut-down. This selection-driven triage accounts for the higher prevalence of male sex, atrial fibrillation and extracardiac arteriopathy in the cut-down group and is the key methodological caveat of our analysis: the strategy was not randomised and the cut-down group represents a higher-risk anatomical subset by design. Despite this allocation bias, the EuroSCORE II and NYHA class were comparable, and the percutaneous group did not show worse outcomes—supporting the safety of percutaneous strategies in appropriately selected patients. We emphasise that because allocation was not randomised, our comparisons reflect the performance of treatment strategies after clinical selection rather than the intrinsic performance of the access techniques themselves.

Our study has additional limitations. It is a retrospective single-centre analysis, the choice of MANTA versus ProGlide was at the surgeon’s discretion, and we did not collect long-term follow-up imaging of the femoral access site (e.g., duplex ultrasound at 6–12 months). Late access-site complications characteristic of large-bore closure—asymptomatic femoral artery stenosis, delayed pseudoaneurysm, plug-related thrombosis and late arterial occlusion—may remain clinically silent and would not be captured within our 30-day window in the absence of systematic duplex surveillance; our complication rates may therefore underestimate the true mid-term burden, and prospective studies with protocolised imaging at 6–12 months are needed. A further limitation concerns the characterisation of the hostile anatomy subset: although preoperative CT angiography and duplex ultrasound informed access selection, the underlying vascular parameters—minimum common femoral artery diameter, anterior wall and circumferential calcification, and iliofemoral tortuosity—were assessed qualitatively at the time of surgery and were not recorded systematically. We are therefore unable to quantify the anatomical differences between the percutaneous and cut-down cohorts beyond the significantly higher prevalence of extracardiac arteriopathy in the latter. This constrains our ability to demonstrate the degree of anatomical triage and reinforces that comparisons between percutaneous access and cut-down reflect treatment strategies after clinical selection rather than the intrinsic performance of the techniques; prospective studies should record protocolised iliofemoral measurements at baseline. The relatively low number of MANTA cases (*n* = 107) may have limited statistical power for some secondary endpoints. A further unmeasured confounder is the difference in arterial cannula calibre among the groups: the mean cannula size was smaller in the MANTA cohort (17.4 ± 1.3 Fr) than in the ProGlide (18.5 ± 1.2 Fr) or cut-down (18.9 ± 1.3 Fr) cohorts. A smaller arteriotomy is intrinsically easier to seal, and part of MANTA’s higher primary haemostasis may therefore reflect cannula size rather than the device’s performance alone; this should be considered when interpreting the inter-device comparison. We also note that, by definition, “primary haemostasis” in the cut-down arm refers to successful open suture closure and is not directly comparable in mechanism with percutaneous device-based closures; the cut-down rate is reported for completeness rather than as a head-to-head benchmark.

Cost-effectiveness data, patient-reported outcomes and quality-of-life metrics were not captured. Prospective, ideally randomised, multicentre trials specific to MICS populations are needed to confirm whether the observed higher rate of primary haemostasis with MANTA translates into clinically meaningful long-term benefit.

## 5. Conclusions

In this single-centre retrospective analysis of 445 consecutive endoscopic MICS patients, percutaneous femoral cannulation was associated with fewer intervention-requiring groin complications and shorter operative times than surgical cut-down, with comparable in-hospital mortality. Within the percutaneous arms, MANTA was associated with a higher rate of primary haemostasis than ProGlide in our institutional experience, while conversion to cut-down rates were equivalent. Surgical cut-down remains an indispensable backup strategy for patients with a hostile femoral anatomy and should not be regarded as inferior but rather as the appropriate choice for a deliberately selected subgroup. The choice of percutaneous closure system should be individualised according to vessel anatomy, institutional experience and economic considerations.

## Figures and Tables

**Table 1:** (**a**) Baseline patient characteristics, percutaneous (*n* = 326) versus cut-down (*n* = 119). (**b**) Multivariable logistic-regression model identifying baseline characteristics associated with closure-device assignment (MANTA versus ProGlide).

(**a**)			
**Variable**	**Percutaneous (*n* = 326)**	**Cut-Down (*n* = 119)**	***p*** **-Value**
Age, years (mean ± SD)	62.2 ± 11.6	59.9 ± 10.4	0.047
Male sex, *n* (%)	186 (57.0)	85 (71.4)	**0.006**
BMI, kg/m^2^ (mean ± SD)	25.4 ± 4.5	26.6 ± 5.4	**0.032**
EuroSCORE II, % (mean ± SD)	1.89 ± 2.55	2.03 ± 2.41	0.594
NYHA class (mean ± SD)	2.51 ± 0.72	2.63 ± 0.59	0.079
Arterial hypertension, *n* (%)	171 (52.4)	45 (37.8)	**0.007**
Diabetes mellitus, *n* (%)	19 (5.8)	7 (5.8)	1.000
Atrial fibrillation, *n* (%)	115 (35.2)	54 (45.3)	0.061
Extracardiac arteriopathy, *n* (%)	31 (9.5)	19 (15.9)	0.063
COPD, *n* (%)	53 (16.2)	17 (14.2)	0.662
Previous stroke, *n* (%)	32 (9.8)	9 (7.5)	0.580
Coronary artery disease, *n* (%)	51 (15.6)	18 (15.1)	1.000
(**b**)			
**Variable**	**Odds Ratio**	**95% CI**	***p*** **-Value**
Age (per year)	1.00	0.97–1.02	0.848
Male sex	2.19	1.13–4.25	**0.021**
BMI (per kg/m^2^)	0.99	0.93–1.06	0.866
EuroSCORE II (per unit)	0.82	0.67–1.00	**0.046**
Hypertension	1.02	0.57–1.81	0.955
Diabetes	0.42	0.11–1.64	0.213
Hyperlipidaemia	1.45	0.79–2.65	0.230
Smoking	0.89	0.46–1.70	0.720
Extracardiac arteriopathy (PAD)	0.87	0.33–2.31	0.778
COPD	0.91	0.43–1.94	0.817
Atrial fibrillation	1.19	0.64–2.20	0.591
Coronary artery disease	1.08	0.51–2.29	0.844
LVEF (per %)	1.01	0.98–1.05	0.461
Model discrimination (C-statistic)	**0.70 *n* = 326 (107 Manta/219 Proglide)**		

*SD, standard deviation; BMI, body mass index; NYHA, New York Heart Association; COPD, chronic obstructive pulmonary disease. OR, odds ratio; CI, confidence interval; BMI, body mass index; LVEF, left ventricular ejection fraction; PAD, peripheral arterial disease; COPD, chronic obstructive pulmonary disease. The model estimates the association of each covariate with closure device assignment (MANTA = 1 vs. ProGlide = 0), not with a clinical outcome; multivariable n = 325 complete cases (107 MANTA/219 ProGlide); model C-statistic (AUC) = 0.70. Bold denotes significance p < 0.05. The two cohorts are only moderately separable on measured baseline/anatomical characteristics (C-statistic = 0.70).*

**Table 2:** (**a**) Intraoperative data across the three access groups. (**b**) Intraoperative procedures performed across the three access groups.

(**a**)				
**Variable**	**ProGlide (*n* = 219)**	**MANTA (*n* = 107)**	**Cut-Down** **(*n* = 119)**	***p*** **-Value**
Cross-clamp time, min	131 ± 42	112 ± 32	163 ± 53	**<0.001**
CPB time, min	201 ± 64	177 ± 50	233 ± 66	**<0.001**
Total operative time, min	266 ± 84	237 ± 62	315 ± 82	**<0.001**
Femoral arterial cannula size, Fr	18.3 ± 1.3	17.7 ± 1.26	18.9 ± 1.3	—
(**b**)				
**Procedure**	**Proglide (*n* = 219)**	**Manta (*n* = 107)**	**Cut-Down (*n* = 119)**	**Total (*n* = 445)**
**Isolated mitral valve procedures**	**187 (85.4%)**	**81 (75.7%)**	**110 (92.4%)**	**378 (84.9%)**
Mitral valve repair	156 (71.2%)	71 (66.4%)	97 (81.5%)	319 (71.7%)
Mitral valve replacement	31 (14.2%)	11 (10.2%)	13 (10.9%)	55 (12.4%)
**Isolated tricuspid valve procedures**	**6 (2.7%)**	**1 (0.9%)**	**2 (1.7%)**	**9 (2.0%)**
Tricuspid valve repair	4 (1.8%)	1 (0.9%)	1 (0.8%)	6 (1.3%)
Tricuspid valve replacement	2 (0.9%)	0 (0.0%)	1 (0.8%)	3 (0.7%)
**Isolated aortic valve procedures** **Combined mitral and tricuspid procedures**	**0 (0.0%)** **26 (11.9%)**	**8 (7.5%)** **17 (15.9%)**	**0 (0.0%)** **7 (5.9%)**	**8 (1.8%)** **50 (11.2%)**
**Concomitant procedures (overall cohort)**				
**Atrial ablation**	42 (19.2%)	27 (25.2%)	41 (34.5%)	110 (24.7%)
**Left atrial appendage closure (LAA)**	24 (10.9%)	25 (23.4%)	17 (14.3%)	66 (14.8%)

*CPB, cardiopulmonary bypass. Values are the mean ± SD. Post hoc Mann–Whitney U tests confirmed significant pairwise differences for cross-clamp, CPB and operative times (all p < 0.005). Bold denotes Main operative categories, or significance.*

**Table 3:** (**a**) Access-site outcomes by closure strategy. (**b**) Multivariable logistic regression model for primary haemostasis (MANTA versus ProGlide), adjusted for arterial cannula size.

(**a**)				
**Variable**	**ProGlide (*n* = 219)**	**MANTA (*n* = 107)**	**Cut-Down (*n* = 119)**	***p*** **-** **Value**
Primary haemostasis, *n* (%)	182 (83.1)	103 (96.2)	119 (100)	**<0.001**
Need for adjunctive device, *n* (%)	29 (13.2)	0 (0.0)	1 (0.8)	**<0.001**
Conversion to cut-down, *n* (%)	8 (3.6)	4 (3.7)	n.a.	1.000
Intervention-requiring groin complication, *n* (%)	8 (3.6)	4 (3.7)	9 (7.5)	0.232
Lymphatic fistula/seroma, *n* (%)	0 (0.0)	0 (0.0)	5 (4.2)	**0.002**
Wound infection, *n* (%)	0 (0.0)	0 (0.0)	1 (0.8)	0.51
(**b**)				
**Variable**	**Odds Ratio**	**95% CI**	***p*** **-Value**	
MANTA (vs. ProGlide)	4.79	1.64–13.95	**0.004**	
Arterial cannula size (per +1 Fr)	0.86	0.67–1.10	0.226	

*Primary haemostasis is defined as effective closure without any need for an adjunctive device or cut-down conversion. n.a., not applicable. The p-values reflect three-group comparisons (χ^2^ or Fisher’s exact test). OR, odds ratio; CI, confidence interval. Reference group: ProGlide. Outcome: primary haemostasis (success of the closure device alone without an adjunctive or second closure device); MANTA n = 107 (103 events, 96.2%) versus ProGlide n = 219 (182, 83* *.1%). Unadjusted MANTA OR = 5.24 (95% CI: 1.82–15.1); Mantel–Haenszel cannula-adjusted OR = 4.62. Bold denotes p < 0.05.*

**Table 4:** Postoperative outcomes (in-hospital and at 30 days).

Outcome	Percutaneous (*n* = 326)	Cut-Down (*n* = 119)	*p*-Value
In-hospital mortality, *n* (%)	7 (2.1)	2 (1.6)	1.000
30-day mortality, *n* (%)	7 (2.1)	2 (1.6)	1.000
Re-thoracotomy for bleeding, *n* (%)	28 (8.5)	5 (4.2)	0.152
New pacemaker implantation, *n* (%)	17 (5.2)	3 (2.5)	0.305
Myocardial infarction/PCI, *n* (%)	2 (0.6)	1 (0.8)	1.000

*PCI, percutaneous coronary intervention.*

## Data Availability

The data underlying this article will be shared upon reasonable request to the corresponding author.
